# Biological Control of *Aedes albopictus*: Obtained from the New Bacterial Candidates with Insecticidal Activity

**DOI:** 10.3390/insects11070403

**Published:** 2020-06-29

**Authors:** Handi Dahmana, Masse Sambou, Didier Raoult, Florence Fenollar, Oleg Mediannikov

**Affiliations:** 1 IRD, AP-HM, MEPHI, Aix Marseille University, 13005 Marseille, France; handi.dahmana@etu.univ-amu.fr (H.D.); didier.raoult@gmail.com (D.R.); 2 IHU-Méditerranée Infection, 13005 Marseille, France; florence.fenollar@univ-amu.fr; 3 IRD, AP-HM, SSA, VITROME, Aix Marseille University, 13005 Marseille, France; massezorro1@gmail.com; 4 Campus Commun UCAD-IRD of Hann, Dakar 18524, Senegal

**Keywords:** mosquito borne diseases, biological control, soil bacteria, secondary metabolites, insecticide

## Abstract

Vector-borne deadly pathogens cause more than 700,000 deaths annually. They are transmitted by several vectors, among which the mosquito is the most important. Chemical compounds often have devastating side effects, leading to the abandonment of the majority of them. Biological control has been performed by using formulations of *Bacillus sphaericus* and *Bacillus thuringiensis,* but their intensive use has led to the emergence of resistance. Currently, the development of new alternative molecules is urgently needed, in order to use them in mosaics or in rotation with already known insecticides for the control of vectors, especially mosquitoes. Here, we attempted to identify bacterial species with potential anti-mosquito actions. Among bacterial strains isolated from dry sandy soil from Senegal, eleven strains from the Bacillales and Actinomycetales orders were chosen for the entomopathogenic activity experiments. Then, we tested their secondary metabolites, which were obtained from the supernatant fraction, and their cell wall and cytoplasmic compounds, which were found in the pellet fraction, in *Aedes albopictus* larvae, and compared the larval mortality rate with that obtained by using a commercial product. A total of 4/11 (36.36%) of the isolated species exhibited insecticidal activity. *B. nealsonii*, which is not a well-known bacterium, had the highest larvicidal effect with 70% of the larval mortality, which is highlighted for the first time. The *Streptomyces* species we isolated seem to be potential new species, and 3/5 (60%) of them exhibited insecticidal activity. Our study reports provide potential candidates for the identification of active molecules to be developed for strengthening the biological control of infectious diseases agents transmitted by mosquitoes.

## 1. Introduction

Vector-borne deadly diseases account for more than 17% of all infectious diseases; among them, 700 million people are infected and more than a million die each year from mosquito-borne illness. Dengue fever is one of the most threatening and up to 4 billion people in more than 128 countries are at risk, which results in an estimated 96 million cases annually [1,2,3,4]. Malaria remains the greatest killer, although great progress has been made in combating malaria in the past two decades; however, its agents still result in millions of cases and thousands of deaths annually (more than 193 million cases in 2017) [5]. Ticks, black flies, sandflies, midges, fleas and triatomine bugs are important vectors of pathogens affecting humans [6], but mosquitoes are still the most well known and most dangerous vector of devastating pathogens.

Dangerous pathogens are transmitted by *Aedes* mosquitoes, such as dengue, Zika and chikungunya, which have accumulated very significant gains in recent years, while old threats, such as yellow fever and Japanese encephalitis, have re-emerged [7,8]. A large number of chemical compounds are used to control them. However, the heavy use of these chemical products has led to several dramatic drawbacks, such as the contamination of water and food sources, the poisoning of nontarget fauna and flora, their concentration in the food chain and emergence of insecticide resistance [9]. When compared to the control strain (not exposed), several studies reported the insecticide susceptibility decrease in *Aedes* sp. associated to resistance development reaching 23% reduction in mortality observed after six generations and around 77% after eight generations [10]. Because of this, the research community has turned away from these molecules and has instead committed to biological control.

More than a thousand naturally occurring microorganisms have been identified as potential insecticidal agents so far, and secondary metabolites from 942 microbial isolates were screened for their insecticidal properties, most of which were bacteria from the *Bacillus* and *Streptomyces* genera [11,12]. In fact, formulations of live *B. sphaericus* and *B. thuringiensis* bacteria are the predominant nonchemical products [13]. However, their intensive use has led to the frequent occurrence of resistance [14,15]. That is why numerous studies are continuously conducted to isolate large collections of *Bacillus* or *Streptomyces* strains to examine their biological activities, including antibacterial, insecticide and other activities [16,17,18,19,20,21,22,23,24].

In our study, we performed the isolation of bacterial strains from a dry sandy soil in Senegal and targeted mostly *Bacillus* and *Streptomyces* species as potential candidates, and we subsequently tested fractions of the supernatant and the pellet against *Aedes albopictus* larva. Metabolites derived from wild bacteria may contain a very large number of molecules with unknown properties, including those with insecticidal effects like those that we are seeking in this study. We aimed to find new bacterial candidates that may be used for the development of insecticidal tools for the biological control of infectious diseases agents transmitted by vectors.

## 2. Materials and Methods

### 2.1. Soil Sampling

**Figure 1:**
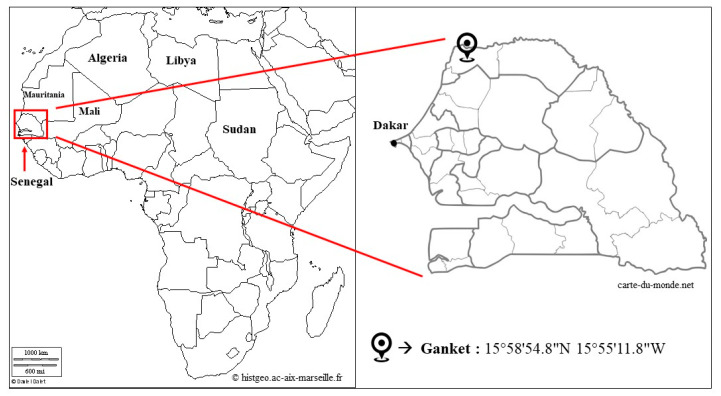
Site of sandy soil sampling in Ganket village, Senegal.

Approximately 30 g of sandy soil (Figure 1) was collected with a sterile spatula at a depth of approximately 10 cm. The sample was then placed in a sterile urine collection vial and stored at room temperature during transport. The samples were stored at −80 °C in (IHU-Méditerranée Infection, Marseille, France) until further experiments.

### 2.2. Bacterial Isolation

The sample was thawed immediately before isolation, homogenized (total homogenization by shaking) and then two times 1 g was taken and mixed in 10 mL of sterile distilled water. Then, the contents were incubated for 24 h at room temperature. Thereafter, the supernatant was recovered and homogenized, and ten serial 1/10 dilutions were performed. The inoculum (50 μL) was seeded on Columbia agar supplemented with 5% sheep blood (bioMérieux, Marcy l’Etoile, France) in actinomycetes isolation agar (Merck, Darmstadt, Germany) and then incubated under aerophilic conditions at 32 °C for at least 48 h for *Bacillus* spp. isolation and for at least 1 week for *Streptomyces* spp. isolation.

### 2.3. Strains Identification

Bacterial species were directly identified from each bacterial colony using matrix-assisted laser desorption ionization-time mass spectrometry (MALDI-TOF MS) (Bruker Daltonics, Bremen, Germany) as previously described [25]. A score of >2 allowed identification at the species level, and a score of <1.7 did not allow any identification. In this case, 16S rRNA gene was amplified and the amplicon sequenced. Briefly, DNA extraction was performed using EZ1 DNA kits (Qiagen, Courtaboeuf, France), according to the manufacturer’s protocol. Amplification and sequencing were performed as described in the study by Dahmana et al. [26] using the 16S universal primers [27].

To obtain the MALDI-TOF spectra of *Streptomyces* species, first, treatment of hard stained colonies on agar was performed with Tissue Lyser (QIAGEN, MD, USA) and tungsten beads for at least 3 min to obtain isolated bacteria, which were suspended in distilled water. Liquid cultures (5 mL) were then generated using the bacterial suspension. Two milliliters of liquid culture were centrifuged at 17,000× *g* for 5 min. The supernatant was discarded and the bacterial pellets were recovered. Subsequently, we performed the extraction of proteins by using an ethanol–formic acid extraction procedure [28]. Briefly, we aliquoted 300 μL of the suspended bacteria in distilled water and added 900 μL ethanol. Subsequently, the cell suspension was centrifuged at 17,000× *g* for 2 min and the supernatant discarded. The centrifugation was repeated and the residual ethanol discarded. The pellet was air-dried and thoroughly resuspended in 5 to 50 μL formic acid–water (70:30 [vol/vol]), depending on its size, and finally, an equal volume of acetonitrile was added. After centrifugation at 17,000× *g* for 2 min, 1 μL of the supernatant was transferred to a polished steel MSP 96 target plate (Bruker Daltonics) and allowed to dry at room temperature before being overlaid with 1 μL of a saturated a-cyano-4-hydroxy-cinnamic acid (HCCA) matrix solution in 50% acetonitrile–2.5% trifluoroacetic acid (Bruker Daltonik). Finally, the matrix sample was crystallized by air-drying it at room temperature for 5 min.

The spectra generated by MALDI-TOF MS without initial identification were recovered to control their quality. After validation of the spectra by using the Bruker software, the MALDI Biotyper 3.0 software was used to build dendrograms that allowed the comparison of the different isolates. The 16S rRNA-based phylogenetic reconstruction does not guarantee well-resolved and robust trees that reflect the overall relationship between *Streptomyces* species, accordingly, to identify the *Streptomyces* strains, the 23S rRNA [29] and *atpD* [30] genes were amplified, and the amplicons were sequenced as described in the study by Dahmana et al. [26]. The electropherograms obtained were assembled and edited using ChromasPro 1.7.7 software (Technelysium Pty Ltd., Tewantin, Australia) and the sequences obtained were compared with those available in the GenBank database by NCBI BLAST (http://blast.ncbi.nlm.nih.gov/Blast.cgi). For taxonomic analyses, the maximum-likelihood phylogenetic trees were constructed using MEGA software version 7.0.21 [31] with 100 bootstrap replications and the species position confirmed using Topali software version V2.5 (2.5.13.04.03) [32]. More specific information is found in the figures.

### 2.4. Fractions Preparation

The strains were stored at −80 °C. The cultures were grown on solid Columbia agar medium with 5% sheep blood (bioMérieux) under aerobic conditions at 32 °C. Thereafter, several colonies were transferred in sterile conditions into 1 L of liquid Tryptic soy broth medium (Sigma-Aldrich, France), which was incubated for 3 days for *Bacillus* spp. and for 14 days for *Streptomyces* spp. at 32 °C and 110 rpm in a shaker incubator under aerobic conditions.

The supernatant-pellet separation was carried out by centrifugation at 8000× *g* for 20 min at 4 °C using an A98813 J-Lite PP bottle assembly with a JLA-8.1000 rotor (Beckman Coulter, Villepinte, France). After centrifugation, the supernatant was immediately filtered through a 0.45 μm filter and placed into 75 mL flasks, after which it was frozen horizontally overnight at −80°C and then lyophilized the next day. The lyophilizate was stored at −20°C prior to the assays.

### 2.5. Release of Inclusions and the Main Cell Components

After centrifugation, the pellet was resuspended in PBS and 700 μL of the resuspension was distributed into 2 mL cryotubes (Bio-One GmbH, Rainbach im Mühlkreis, Austria). In order to exclude the effects of live bacteria on future experiments, we disintegrated the bacteria by consecutive freezing/thawing and sonication treatments. Each tube was subjected to three freeze–thaw cycles for 5 min each using liquid nitrogen and a hybridization incubator heated to 50 °C. The tubes were centrifuged at 13,000× *g* for 10 s, and the contents were transferred into 1.5 mL Eppendorf Safe-Lock tubes (Eppendorf, Montesson, France) and then subjected to 3 sonication cycles at an amplitude of 50 Hz for 30 s. Subsequently, ultracentrifugation was carried out at 20,000× *g* for 20 min at 4 °C. Thereafter, the supernatant was recovered, directly filtered through a 0.45 μm filter and stored at −20 °C prior to the assays. All fractions were regularly cultured on Columbia sheep blood agar plates (bioMérieux) after filtration to ensure their sterility.

### 2.6. Fractions Used in Larval Assays

Once the larvae were ready (third and early fourth instar), the sterile fractions (pellet and supernatant) already prepared of a bacteria strain were thawed at room temperature, after which the Bradford protein assay (BIO-RAD, Schiltigheim, France) was carried out. Subsequently, the volume of the fraction used was adjusted to have two concentrations, 2 and 6 mg/L, which were administered to the larvae. We tested the supernatant and pellet fractions of each stain separately at concentrations of 2 and 6 mg/L. We also tested the mixture of each of the two fractions at 6 mg/L to determine if there was a synergetic effect of the secreted and cell constituent compounds.

We used *Bacillus thuringiensis* subsp. *israelensis* AM65-52 (*Bti*), which was isolated from a commercial granular formulation (VectoBacGR, Valent Bioscience, Libertyville, IL, USA), to validate our protocol and to assess the insecticidal activity of the strain used as a positive control. Thus, in both the studied bacterial strains and in positive controls, we eliminated the direct effect of live bacteria on mosquito larvae (only sterile fractions were tested). We administered the bacterial fractions 24 h before feeding the larvae to give them enough time to ingest the bacterial fraction compounds.

### 2.7. Screening for Insecticidal Activity

The *Aedes albopictus* laboratory colony was maintained at 27 ± 0.5 °C and in 80 ± 5% relative humidity. Adult mosquitoes were maintained with constant exposure to 10% sterile sucrose on cotton balls that were changed daily. For egg production, adult female mosquitoes were given defibrinated human blood (French Blood Agency, France) via the Haemotek membrane feeding system (Haemotek Ltd., Blackburn, UK). Larvae were fed Tetra-Min (Spectrum Brands, Fennimore, WI, USA) fish food in clear water until the pupae stage.

Seventy-five milliliter flasks were used for the insecticide screening assays. Only third and early fourth instar larvae were used in the insecticidal activity assays. All tests of each fraction were performed with 25 larvae (N = 4), and a total of 100 larvae for each fraction were tested as recommended by the WHO [33]. Immediately after separating the larvae in flasks containing 100 mL of clean distilled water, we added the calculated volume of insecticide. Larvae were not fed until the 24th hour. Dead larvae were counted at 24, 48 and 72 h. In each assay, 100 larvae were used as negative controls, which did not receive any fraction, to assess natural mortality. We considered that a strain had a good insecticidal activity if it induced >20% mortality.

### 2.8. Data Analysis

We used the Epi Info version 7 program (http://www.cdc.gov/epiinfo/index.html) (Addinsoft, 2019) and the XLSTAT statistical and data analysis solution (Paris, France, https://www.xlstat.com) to perform the statistical analyses. The Kruskal–Wallis test, the comparison of k-proportions test and the pairwise comparison test were performed to compare the mortality rates recorded at 72 h after administration of 6 mg/L of each supernatant fraction. A difference was statistically significant when the *p*-value was ≤0.05. The Dunn procedure (bilateral test) was performed to separate groups of strains according to their efficiencies against *A. albopictus* larva. The total number of living *A. albopictus* larvae was transformed to the arithmetic means. The insecticidal effects were calculated at 72 h for each active fraction using Abbott’s formula (Abbott, 1987) as follows:

$$Insecticidal\;efficacy\;\left(\%\right)=100*\frac{NCi-NTi}{NCi}.$$

where *NCi* and *NTi* represent the geometric mean (GM) of living *A. albopictus* in the control and treated groups, respectively.

## 3. Results

### 3.1. Isolated Strains

**Figure 2:**
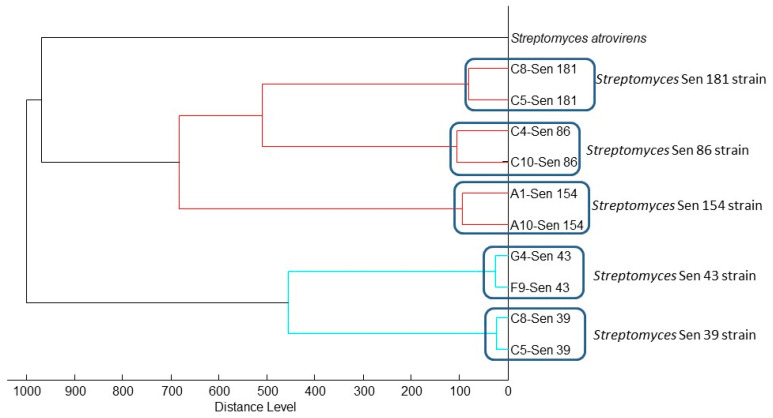
Dendrograms for the *Streptomyces* strains generated using the MALDI Biotyper 3.0 software, with *Streptomyces atrovirens* as the outgroup.

**Figure 3:**
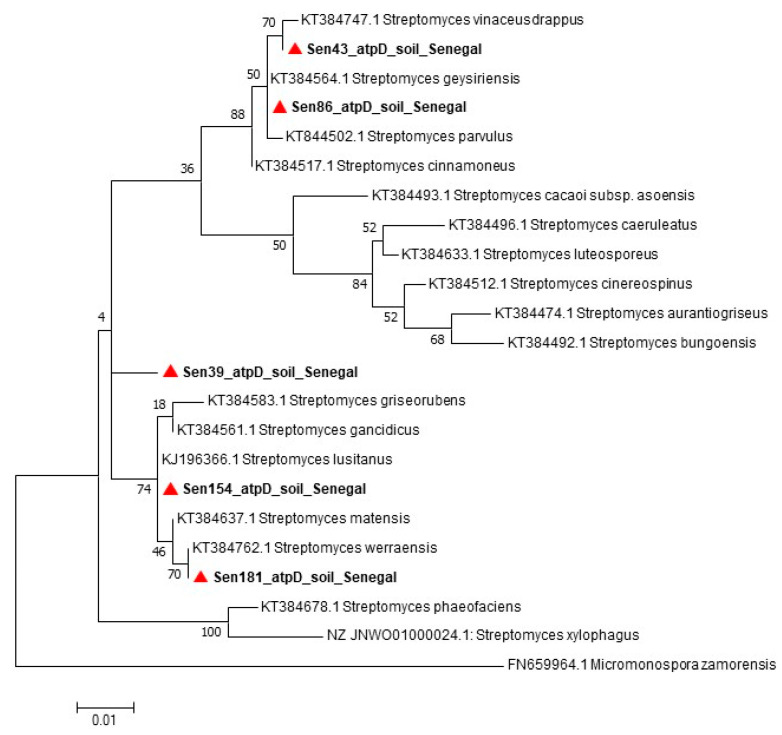
Maximum-likelihood phylodendrogram of *Streptomyces* spp., including the species isolated in the present study based on the partial 466-bp sequence of the *atpD* gene.

**Figure 4:**
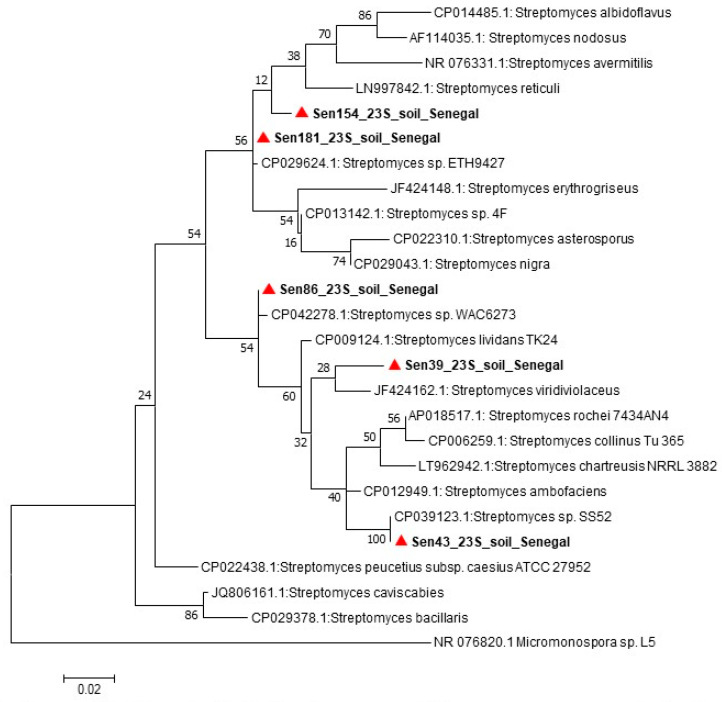
Maximum-likelihood phylodendrogram of Streptomyces spp., including the species isolated in the present study based on the partial 1000-bp sequence of the 23S rRNA gene.

Four *Bacillus* strains were directly identified by MALDI-TOF MS, while the strain Sen140 was not identified by mass spectrometry, although a good quality spectrum was obtained that did not match any spectra in the MALDI-TOF database. Subsequently, for the strain Sen140, the 16S gene was sequenced and deposited in GenBank (accession number: MN788519). *Streptomyces* strains could not be identified by MALDI-TOF either by the direct method or by the direct ethanol–formic acid extraction method [34]. Using the optimized protocol (see above), good-quality spectra were obtained (Figure S1), but identification could not be achieved because of the lack of *Streptomyces* spectra in the database. The dendrogram built using MALDI Biotyper 3.0 software permitted the comparison of the different *Streptomyces* spectra (Figure 2). The analysis of the sequences of the *atpD* (Figure 3) and 23S rRNA (Figure 4) genes showed that the *Streptomyces* strains isolated in this study may represent potential new species.

The accession numbers of the 23S and *atpD* genes were obtained and are shown in Table S2. For the following experiments, we chose bacteria from the *Bacillus*, *Brevibacillus*, *Micrococcus* and *Streptomyces* genera representing 11 strains (Table 1).

### 3.2. Screening for Insecticidal Activity

A total of 108 strains from 15 different genera were isolated. Because Actinomycetales and Bacillales are known for their production of compounds exhibiting entomopathogenic activity, we focused our study on strains belonging to both of these orders, corresponding to eleven strains that were chosen for the entomopathogenic activity experiments. The secondary metabolites of these strains were produced, concentrated and then screened for insecticidal activity. The overall results are presented in Table 2. We noticed for all strains that insecticidal activity caused by the main cell components was absent and that mosquito larvae mortality caused by the supernatants was dose-dependent. When tested at a concentration of 6 mg/L, supernatants from 36.4% (4/11) of the bacterial strains exhibited activity against the *A. albopictus* larvae. Regarding negative control and throughout the testing periods, no larva was found dead.

The most active species was *Bacillus nealsonii* Sen 132, which resulted in 70% larval mortality that was increased to 84% when it was mixed with the pellet fraction. The activity of the other species was as follows: *Streptomyces* sp. Sen 86 resulted in 41% larval mortality, *Streptomyces* sp. Sen 39 resulted in 36% larval mortality and *Streptomyces* sp. Sen 154 resulted in 31% larval mortality. We noticed that for most of the species, the rate of larval mortality did not change considerably when the supernatant was combined with the pellet fraction (Table 2). Among the rest of the species, 63.6% (7/11) were not active or had the lowest activity against the mosquito larvae, resulting in ≤20% mortality (Table 2). When comparing the insecticidal activity of the supernatant fractions from those of the different strains at 72 h with 6 mg/L, the difference was significant (critical value of khi^2^ = 19,675, *p*-value ≤ 0.0001). The comparison of the insecticidal activity performed included the reference strain (Table S1).

The efficacy of the strains was compared to that of the *Bti* positive control (Table 3). We used this strain as a control for several reasons. It produces different Cry toxins that, after ingestion, are able to form pores in the plasma membrane of midgut epithelial cells in susceptible insects [35]. Using the Dunn test, the strains were classified into four groups according to their efficiencies (Paris, France, https://www.xlstat.com). Group D was the most effective, as it was composed of only the *B. nealsonii* species, which had a mortality rate more than twice that of the positive control, with a mean rank equal to 887.5. Group C, which was composed of the three other active species and the positive control, had a mortality rate ranging from 31% to 41%, and a mean rank ranging from 647.5 to 713.5. The B and C groups were mostly comparable, while group A was the least effective and was composed of the strains with the lowest activity and effectiveness (Table S1), and with mean ranks ranging from 479.5 to 575.5.

The insecticidal activity of only one species, *B. nealsonii* Sen 132, was significantly increased, and it killed more than 2/3 of *A. albopictus* larval, whereas the reference strain *Bti* killed only 33%. Furthermore, *B. nealsonii* Sen 132 was much more active compared to the other strains in group A isolated in this study.

## 4. Discussion

A total of 108 strains from 15 different genera were isolated. Because Actinomycetales and Bacillales are known for their production of compounds exhibiting entomopathogenic activity, we focused our study on testing strains belonging to both of these orders. Eleven strains were tested for their insecticidal activity by using *A. albopictus* larvae. Insect-borne diseases cause hundreds of thousands of deaths in humans every year, and mosquitoes such as *Aedes* species are among the most important vectors of these deadly pathogens [12,36,37,38].

The search for new insecticidal molecules is a global need due to the re-emergence of deadly disease vectors, the control of which depends on identifying new insecticidal molecules [38]. The widespread emergence of insecticide resistance complicates this mission concerning essential chemical compounds due to several factors, one being species fitness [39]. Furthermore, we know that such compounds are key for the control of diseases such as malaria, which is currently threatened by resistance development that has been reported in over 80% of countries with endemic malaria [40]. For its various positive points, biological control has become more common and has attracted a great deal of attention [12].

*Streptomyces* are Gram-positive bacteria and are the largest representative genus of actinomycetes, which are found mostly in soil [41]. They are industrially useful microorganisms that produce a wide variety of antibiotics, and several studies have been carried to investigate their biological effects [42,43,44]. Species of the *Bacillus* genus are generally also found in soil, and they have many benefits [45], especially for crop improvement, due to biomolecular changes in adverse environments [46], defense against vectors of infectious diseases [47] and antimicrobial properties [20]. For this purpose, different studies were carried out involving the isolation of bacteria from the soil for biological effects research [20,22,24,45]. A study was carried out in Senegal to search for strains of *B. thuringiensis* and *B. sphaericus* useful for the control of malaria, which revealed that 27/203 (13.3%) of strains were active [48]. Currently, many studies report resistance to the latter species [49,50,51], and we must seek better solutions. We focused on secreted metabolites and the components of bacterial cell walls or cytoplasm. Eleven different strains of *Bacillus* (N = 5), *Micrococcus* (N = 1) and *Streptomyces* (N = 5) were chosen for further studies for the identification of insecticidal activity.

Secondary metabolites were produced and separated into two fractions. The supernatant fraction contained the secreted metabolites and the pellet fraction contained the cell wall and cytoplasmic metabolites. Then, screening for insecticidal activity was carried out and 36.4% (4/11) of the strains showed insecticidal activity that killed more than 20% of *A. albopictus* larvae during testing of the supernatant fraction (given the very small amount of material tested); a mortality greater than 20% is considered promising (the use of a pure compound will give better activity).

Several projects studied large collections of *Bacillus* species to identify direct biological activity, including insecticidal activity [20,21,22,24], and numerous other *Bacillus* spp. show high toxicity against dipterans, such as *Bacillus circulans* [52] and *Brevibacillus laterosporus* [53,54]. Although many *Bacillus* strains were tested previously, it is clear that numerous species remain undiscovered and not officially described [55,56]. *B. nealsonii* was already isolated from potentially hostile environments, including a spacecraft assembly and the rumen of a buffalo [57,58], and it is not well characterized or shown great usefulness except for its very limited application to the production of proteases and as an additive detergent [59,60]. The secondary metabolites of the strain Sen132 of *B. nealsonii* that we isolated were screened for mosquitocidal activity and exhibited high activity against *A. albopictus* larvae. A total of 70% of the third and fourth instar larvae were killed in less than 72 h by the supernatant fraction, showing that these strains secrete a potential insecticidal compound in the culture medium. Further studies are needed to characterize the active compounds of these good bacterial candidates as agents for the biocontrol of mosquitoes, which we have demonstrated for the first time. The other species of the *Bacillus* genus that we tested had no or low larvicidal activity. This may mean that these species do not produce insecticidal molecules or that the optimization of the necessary parameters to ensure their optimal production is required, as our production protocol was standardized for all the bacteria used in this study.

Additionally, the supernatant fractions of three (60%) of the five species of *Streptomyces* that we isolated exhibited good insecticidal activity, which is comparable to that produced by the positive control *Bacillus thuringiensis* subsp. *israelensis* AM65-52 that was isolated from a commercial granular formulation. Numerous studies have already carried out the isolation of *Streptomyces* bacteria from soil to look for potential insecticidal effects on different insects of agronomic or public health interest [17,18,19,23]. Actinobacteria are excellent producers of antibiotics and enzymes, and *Streptomyces* are the most relevant due to their ability to produce a large number of antibiotics, in addition to other classes of biologically active secondary metabolites. Some antibiotics were identified to have insecticidal activities, including respiratory inhibitors such as antimycin A, patulin and piericidins, protein synthesis inhibitors such as cycloheximide or tenuazonic acid, and membrane-active agents such as some polyene macrolide antibiotics [12,61,62]. *Streptomyces* species can play a very important role in the biocontrol of insect pests, especially mosquitoes, by the direct production of active insecticide compounds [63,64,65,66]. Ivermectin is the most well-known insecticide produced by *Streptomyces*, which was initially used as an antiparasitic agent that has become a systemic treatment for the control of arthropod pests in livestock [67].

We used MALDI-TOF mass spectrometry as well as two sensitive and discriminating PCR systems for the identification of the *Streptomyces* species we isolated, which revealed that we isolated potential new species or species with sequences that do not exist in the GenBank database. Therefore, the first step in the objective of discovering which compounds are produced is to completely sequence the whole genomes of these species and then proceed to the characterization of the active molecules. The screening of these species for the production of antimicrobial agents also seems to be a track, which is also in progress.

## 5. Conclusions

In conclusion, we isolated noteworthy bacterial species that exhibit remarkable insecticidal activity compared to that of a commercial product. Among them, this was the first time that *B. nealsonii* was tested against *A. albopictus* larvae, and its supernatant fraction exhibited very high larvicidal activity, which means that its secondary metabolites were secreted in the culture medium. We also isolated several species from the *Streptomyces* genus, of which three were active, and advanced studies are required to characterize the active compounds. Our study reports results that may be still preliminary, but provide potential candidates for the identification of active molecules to be developed for strengthening the biological control of vectors of infectious disease agents transmitted by mosquitoes. It will be very important to test these compounds against other important mosquito species and other insects of medical, veterinary and agricultural interest.

## Figures and Tables

**Table 1:** Isolated strains used in the study.

Order	Genus and Species	Strain Code
*Actinomycetales*	*Streptomyces* sp.	Sen 181
*Actinomycetales*	*Streptomyces* sp.	Sen 43
*Actinomycetales*	*Streptomyces* sp.	Sen 154
*Actinomycetales*	*Streptomyces* sp.	Sen 86
*Actinomycetales*	*Streptomyces* sp.	Sen 39
*Bacillales*	*Brevibacillus brevis*	Sen 108
*Bacillales*	*Bacillus nealsonii*	Sen 132
*Actinomycetales*	*Micrococcus luteus*	Sen 7
*Bacillales*	*Bacillus pumilus*	Sen 186
*Bacillales*	*Bacillus subtilis*	Sen 66
*Bacillales*	*Bacillus* sp.	Sen 140

**Table 2:** Detailed results of the insecticidal activity of the secondary metabolites of the isolated species against third and early fourth instar *A. albopictus* larvae at 72 h post-administration.

Species	Strain	Negative Control	Pellet	Supernatant		Supernatant + Pellet (6 mg/L) *	Notes
			(2–6 mg/L)	2 mg/L	6 mg/L		
*Streptomyces* sp.	Sen 181	0%	0%	0%	18%	20%	No potential Insecticidal activity
*Streptomyces* sp.	Sen 43	0%	0%	0%	12%	8%	No potential Insecticidal activity
*Streptomyces* sp.	Sen 154	0%	0%	29%	31%	28%	Potential Insecticidal activity
*Streptomyces* sp.	Sen 86	0%	0%	35%	41%	32%	Potential Insecticidal activity
*Streptomyces* sp.	Sen 39	0%	0%	30%	36%	40%	Potential Insecticidal activity
*Brevibacillus brevis*	Sen 108	0%	0%	0%	2%	4%	No potential Insecticidal activity
*Bacillus nealsonii*	Sen 132	0%	0%	40%	70%	84%	Potential Insecticidal activity
*Micrococcus luteus*	Sen 7	0%	0%	4%	4%	12%	No potential Insecticidal activity
*Bacillus pumilus*	Sen 186	0%	0%	5%	6%	4%	No potential Insecticidal activity
*Bacillus subtilis*	Sen 66	0%	0%	10%	10%	12%	No potential Insecticidal activity
*Bacillus* sp.	Sen 140	0%	0%	4%	6%	8%	No potential Insecticidal activity
*Bacillus thuringiensis*	AM65-52	0%	0%	15%	33%	34%	Insecticidal activity

*: (6 mg/L) each.

**Table 3:** Comparison of the efficacy of the strains to that of the positive control *Bti*.

Strain	Code	Mortality Rate	Standard Deviation	Groups	Mean Rank	*p*-Value	Significance
***Bti***	**AM65-52**	**33%**	**Ref**	**B-C**	**Ref**	**Ref**	**Ref**
*Streptomyces* sp.	Sen 181	18%	0.386	A-B	90	0.011	Neg. S **
*Streptomyces* sp.	Sen 43	12%	0.302	A	138	≤0.0001	Neg. S **
*Streptomyces* sp.	Sen 154	31%	0.461	B-C	18	0.610	NS ***
*Streptomyces* sp.	Sen 86	41%	0.494	C	−48	0.173	NS ***
*Streptomyces* sp.	Sen 39	36%	0.482	B-C	−18	0.610	NS ***
*Brevibacillus brevis*	Sen 108	2%	0.141	A	186	≤0.0001	Neg. S **
*Bacillus nealsonii*	Sen 132	70%	0.461	D	−222	≤0.0001	Pos. S *
*Micrococcus luteus*	Sen 7	4%	0.197	A	174	≤0.0001	Neg. S **
*Bacillus pumilus*	Sen 186	6%	0.239	A	162	≤0.0001	Neg. S **
*Bacillus subtilis*	Sen 66	10%	0.302	A	138	≤0.0001	Neg. S **
*Bacillus* sp.	Sen 140	6%	0.239	A	162	≤0.0001	Neg. S **

* -Pos. S: Positively significant ** -Neg. S: Negatively significant *** -NS: Non-significant.

## Data Availability

Sequences of sequenced strains are deposited in GenBank. All data generated or analyzed during this study are included in this manuscript and its Supplementary Information Files.

## Supplementary Materials

The following are available online at https://www.mdpi.com/2075-4450/11/7/403/s1, Figure S1: Analyses of the spectra obtained from the *Streptomyces* strains using MALDI Biotyper 3.0 software, Table S1: Dunn pairwise test results. Comparison of insecticidal activity rates between the different isolated species and *Bti* (AM65-52), Table S2: Accession numbers of the sequenced *Streptomyces* species.
