# Supplementary material for: Biological Control of Aedes albopictus: Obtained from the New Bacterial Candidates with Insecticidal Activity
Source: Insects. 2020 Jun 29;11(7):403. doi: 10.3390/insects11070403 (PMC7412510; doi:10.3390/insects11070403)
Supplement: Supplementary file 1 [file insects-11-00403-s001.zip › insects-830369-new/Figure S1.pdf]

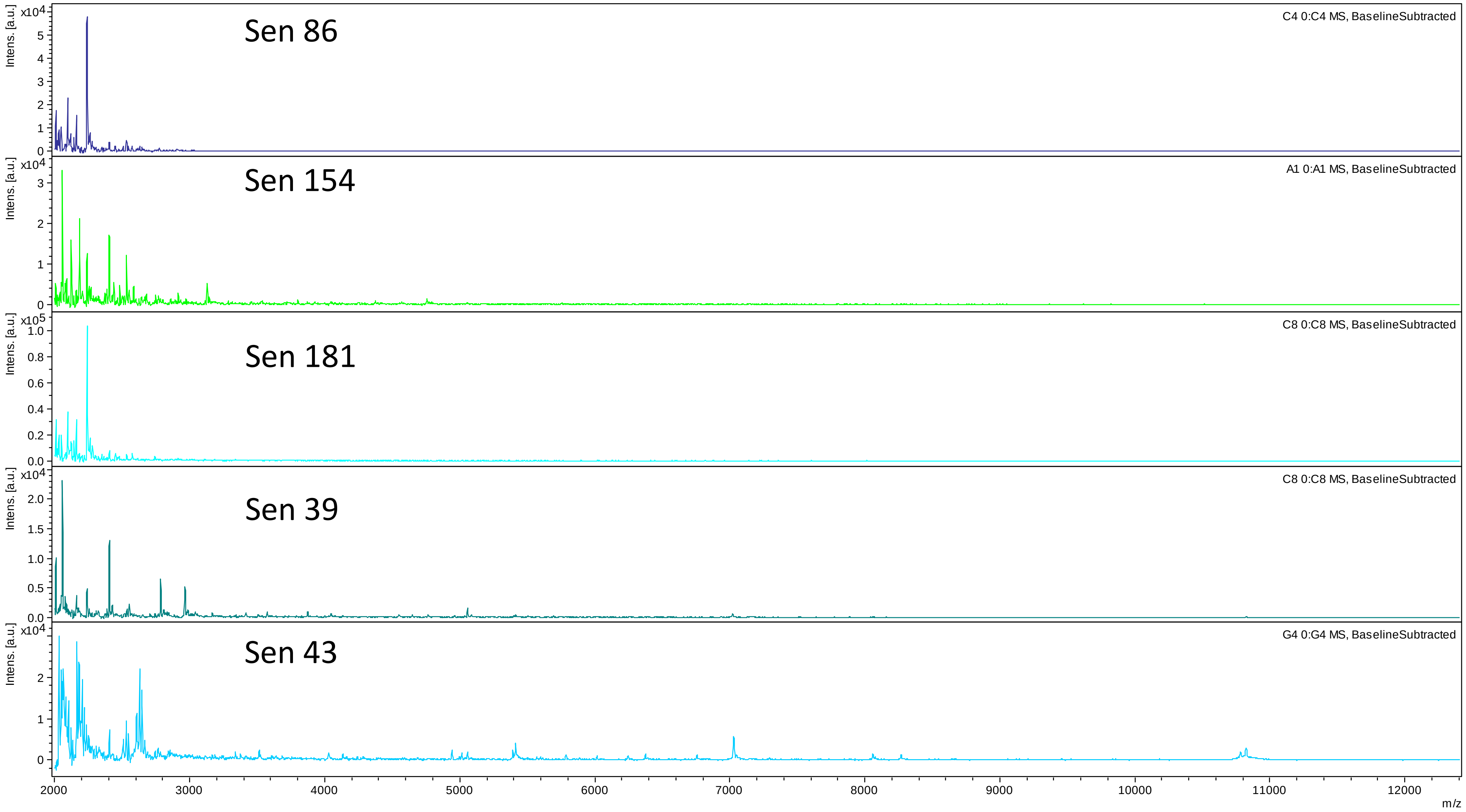

Figure S1. Analyses of the spectra obtained from the *Streptomyces* strains using MALDI Biotyper 3.0 software.
