# Supplementary material for: Biological Control of Aedes albopictus: Obtained from the New Bacterial Candidates with Insecticidal Activity
Source: Insects. 2020 Jun 29;11(7):403. doi: 10.3390/insects11070403 (PMC7412510; doi:10.3390/insects11070403)
Supplement: Supplementary file 1 [file insects-11-00403-s001.zip › insects-830369-new/Table S1.pdf]

Table S1: Dunn pairwise test's results. Comparison of insecticidal activity rates between the different isolated species and *Bti* (AM65-52).

		***Streptomyces* sp Sen 181**	***Streptomyces* sp Sen 43**	***Streptomyces sp Sen 154***	***Streptomyces* sp Sen 86**	***Streptomyces* sp Sen 39**	***Brevibacillus brevis***	***Bacillus nealsonii***	***Micrococcus luteus***	***Bacillus pumilus***	***Bacillus subtilis***	***Bacillus sp.***	***Bti* AM65-52**
	***Streptomyces* sp Sen 181**		**0,173**	**0,041**	**< 0,0001**	**0,002**	**0,006**	**< 0,0001**	**0,017**	**0,041**	**0,173**	**0,041**	**0,011**
	***Streptomyces* sp Sen 43**	**-48,000**		**0,001**	**< 0,0001**	**< 0,0001**	**0,173**	**< 0,0001**	**0,307**	**0,496**	**1,000**	**0,496**	**< 0,0001**
	***Streptomyces* sp Sen 154**	**72,000**	**120,000**		**0,061**	**0,307**	**< 0,0001**	**< 0,0001**	**< 0,0001**	**< 0,0001**	**0,001**	**< 0,0001**	**0,610**
	***Streptomyces* sp Sen 86**	**138,000**	**186,000**	**66,000**		**0,395**	**< 0,0001**	**< 0,0001**	**< 0,0001**	**< 0,0001**	**< 0,0001**	**< 0,0001**	**0,173**
	***Streptomyces* sp Sen 39**	**108,000**	**156,000**	**36,000**	**-30,000**		**< 0,0001**	**< 0,0001**	**< 0,0001**	**< 0,0001**	**< 0,0001**	**< 0,0001**	**0,610**
**Mean rank**	***Brevibacillus brevis***	**-96,000**	**-48,000**	**-168,000**	**-234,000**	**-204,000**		**< 0,0001**	**0,734**	**0,496**	**0,173**	**0,496**	**< 0,0001**
	Bacillus nealsonii	**312,000**	**360,000**	**240,000**	**174,000**	**204,000**	**408,000**		**< 0,0001**	**< 0,0001**	**< 0,0001**	**< 0,0001**	**< 0,0001**
	Micrococcus luteus	**-84,000**	**-36,000**	**-156,000**	**-222,000**	**-192,000**	**12,000**	**-396,000**		**0,734**	**0,307**	**0,734**	**< 0,0001**
	Bacillus pumilus	**-72,000**	**-24,000**	**-144,000**	**-210,000**	**-180,000**	**24,000**	**-384,000**	**12,000**		**0,496**	**1,000**	**< 0,0001**
	Bacillus subtilis	**-48,000**	**0,000**	**-120,000**	**-186,000**	**-156,000**	**48,000**	**-360,000**	**36,000**	**24,000**		**0,496**	**< 0,0001**
	***Bacillus* sp.**	**-72,000**	**-24,000**	**-144,000**	**-210,000**	**-180,000**	**24,000**	**-384,000**	**12,000**	**0,000**	**-24,000**		**< 0,0001**
	***Bti* AM65-52**	**90,000**	**138,000**	**18,000**	**-48,000**	**-18,000**	**186,000**	**-222,000**	**174,000**	**162,000**	**138,000**	**162,000**	
