# Supplementary material for: Biological Control of Aedes albopictus: Obtained from the New Bacterial Candidates with Insecticidal Activity
Source: Insects. 2020 Jun 29;11(7):403. doi: 10.3390/insects11070403 (PMC7412510; doi:10.3390/insects11070403)
Supplement: Supplementary file 1 [file insects-11-00403-s001.zip › insects-830369-new/Table S2.docx]

**Table S2.** Accession numbers of the sequenced *Streptomyces* species.

| **Genus** | **Strain** | **Gene** | **Accession Number** | **Gene** | **Accession Number** |
| --- | --- | --- | --- | --- | --- |
| *Streptomyces* | Sen181 | *23S* | MN617839 | *atpD* | MN626483 |
|  | Sen43 | *23S* | MN617838 | *atpD* | MN626486 |
|  | Sen86 | *23S* | MN617837 | *atpD* | MN626484 |
|  | Sen39 | *23S* | MN617836 | *atpD* | MN626482 |
|  | Sen154 | *23S* | MN617754 | *atpD* | MN626485 |
